# Why the Conjunction Effect Is Rarely a Fallacy: How Learning Influences Uncertainty and the Conjunction Rule

**DOI:** 10.3389/fpsyg.2018.01011

**Published:** 2018-07-04

**Authors:** Phil Maguire, Philippe Moser, Rebecca Maguire, Mark T. Keane

**Affiliations:** ^1^Department of Computer Science, Maynooth University, Maynooth, Ireland; ^2^Department of Psychology, Maynooth University, Maynooth, Ireland; ^3^School of Computer Science and Informatics, University College Dublin, Dublin, Ireland

**Keywords:** conjunction fallacy, learning, probability theory, informativeness, randomness deficiency, surprise, subjective uncertainty, subjective likelihood

## Abstract

In this article we explore the relationship between learning and the conjunction fallacy. The interpretation of the conjunction effect as a fallacy assumes that all observers share the same knowledge, and that nobody has access to privileged information. Such situations are actually quite rare in everyday life. Building on an existing model of surprise, we prove formally that in the more typical scenarios, where observers are alert to the possibility of learning from event outcomes, the conjunction rule does not apply. Scenarios which have been engineered to produce the so-called conjunction “fallacy” (e.g., Tverksy and Kahneman, 1983) often imply subjective uncertainty and hence the possibility of learning. In Experiment 1 we demonstrate that when these scenarios are rephrased so as to eliminate subjective uncertainty, the effect is mitigated. In Experiment 2 we demonstrate that when subjective uncertainty is reduced by allowing participants to learn about the mechanism behind a conjunction-inducing scenario, the conjunction effect again diminishes. We conclude that the conjunction effect arises due to the unnaturalness of interpreting verbal descriptions in terms of a situation in which all observers share the same knowledge. Instead, when people hear descriptions of real world situations, they are likely to assume that learning is possible, and that subjective rather than objective uncertainty applies.

## 1. Introduction

Every day, people deploy their knowledge to carry out a bewildering array of cognitive tasks, ranging from motor tasks to perception, to reasoning and decision making. This knowledge is tenuous. Not only are the facts themselves uncertain, but so too are the assumed causal models that allow facts to be inferred in the first place. At any moment we may be faced by a surprising revelation which requires us to fundamentally re-evaluate our representation of what we thought was the case. As such, our knowledge is not set in stone, but continually modified, resulting in a dynamic and ever changing mental representation of the world.

In this article we highlight the fact that there are two very different types of uncertainty, one we refer to as “objective uncertainty,” which is shared by all observers and does not result in any representational updating, and the other we refer to as “subjective uncertainty,” which is asymmetric, and causes different people to learn in different ways. These two types of uncertainty invoke different sets of probabilistic rules, a divergence which, we will argue, lies behind the conjunction effect. Specifically, most scenarios in everyday life involve subjective uncertainty, for which the conjunction rule does not hold.

### 1.1. Objective uncertainty

Objectively uncertain outcomes are those that involve pure randomness, from which no learning takes place. Each event is independent, and completely unconnected to the next.

When we roll a dice, for example, and observe the result, it does not lead us to update our expectations for future rolls. We already know everything about the dice. Such gambling tools are designed to guarantee that everybody is equally uninformed. Nobody can have privileged knowledge of a fair dice.

Over the last century, probability theory has emerged as the ubiquitous approach for modeling objective uncertainty. At its heart lies the assumption that all observers share the same information: probabilities are expressed relative to a fixed stochastic model, which is available to all observers. The theory was formalized by Kolmogorov in the 1930s through the notion of probability space, whereby a set of possible outcomes is mapped to a number that represents its likelihood by a probability measure function.

In this context, the concept of a repeated experiment, which is central to the frequentist interpretation of probability, is applicable. The uncertainty is *objective* in the sense that there is a fixed stochastic model which processes randomness, and this model can be sampled repeatedly without changing one's beliefs about the model. If we agree with all of these assumptions, then a person who judges the sequence of coin tosses *HTHHTH* as more likely than *HHHHHH* is reasoning fallaciously, since both are equiprobable (Griffiths and Tenenbaum, 2004).

### 1.2. Subjective uncertainty

The types of situations in which probability theory excels are those for which a precise and reliable stochastic model can be identified, leading to high confidence in the assumption of independent outcomes. In other words, probability works for situations involving objective uncertainty, where everybody is equally uncertain as to what will happen next.

In economics and decision-making research, the term “Knightian uncertainty” is used to describe the type of uncertainty which results from reaching a fundamental limit to knowledge (e.g., quantum decay; see Knight, 1921). Such barriers ensure that future events are genuinely unpredictable, and hence that all observers are equally placed to judge outcomes. Knightian uncertainty is necessarily objective, as no observer can get an edge over any other.

In the real world, however, situations involving Knightian (i.e., shared) uncertainty are extremely rare. Instead, people typically expect to *learn* from their observations, with each individual learning at different rates, and holding different levels of insight. Rather than assuming a fixed model of reality, people are sensitive to any patterns that link outcomes together, thereby suggesting the presence of a superior underlying explanatory model (e.g., is there something I don't know that other people might know?; see Schmidhuber, 2009; Maguire et al., 2018). This idea, that outcomes can be *subjectively* informative, clashes with a core assumption of probability theory, namely that successive outcomes should be treated as independent of each other.

When we henceforth use the terms “subjective” and “objective” uncertainty in this paper, we are referring, not to the holder of the uncertainty, but to the issue of whether outcomes are viewed as dependent or independent. Ramsey (1926) for instance, showed that the laws of probability can be satisfied by a “subjective” idiosyncratic belief system. He suggested that the degree of probability that an individual attaches to a particular outcome can be measured by finding what odds they would accept when betting on that outcome. As long as people's internal beliefs remain probabilistically coherent (e.g., the probability of an event and its negation must sum to 1; see Coletti and Scozzafava, 2002), then any such system will work. However, when we use the world “subjective,” we are referring, not to the personalization of probability in terms of belief (e.g., as per Jeffrey's, 2004 presentation of subjective probability), but to the issue of learning from observation. Because it assumes outcome independence, classical probability cannot be used to quantify the likelihood of informative events.

For example, what if a tossed coin repeatedly shows heads 10, 100, or even 1,000 times in a row? Classical probability theory says that 1,000 heads in a row is just as likely as any other particular sequence of 1,000 coin tosses, so no need to do anything. But in the real world, would we really accept this? Would we be so confident as to maintain the assumption of independence between coin tosses? The greater the deviation from a typically random sequence, the more our faith in the neutrality of the coin is tested. We know that somebody might be tricking us. A repeated pattern of heads appears to leak subjective information about some bias in the coin. This lack of absolute faith in outcome independence reflects a source of “subjective uncertainty” that is incompatible with classical probability theory.

Even generative mechanisms which are strongly believed to be random can produce sequences that expose latent subjective uncertainty, and lead people to question the assumption of independent outcomes. Such an event occurred in 2009 in the Bulgarian national lottery, when the same set of six numbers was drawn in two consecutive draws. An unprecedented record number of 18 people shared the winnings from the second draw, having correctly anticipated a repeat of the previous week's numbers. The officials of the lottery insisted that manipulation was impossible and that it must have been a freak co-incidence. Nevertheless, the Bulgarian minister of Physical Education and Sport established a commission to investigate the incident, indicating a lack of complete confidence in the randomness of the draw. Maybe the balls were not equally weighted, maybe the drum mechanism was defective, or maybe the lottery officials were corrupt. In sum, the occurrence of the same set of numbers on two consecutive occasions was so surprising that it served to undermine confidence in the assumption of objective uncertainty.

In classical probability theory, the probability distributions themselves remain invariant. However, in the case of subjective uncertainty, observers learn from outcomes and update their expectations accordingly. The invariant in this case is a system for extracting and processing the information derived from observations. Adherence to a fixed stochastic model (i.e., assuming outcomes are independent of each other) precludes any possibility of learning, a disposition which Baldi and Itti (2010) argue would be detrimental to survival if applied in the real world. In contrast, observers have to be ready to change their beliefs, which means they need to be receptive to the signals of leaked subjective information, a phenomenon which has been referred to as “surprise.”

## 2. Surprise

Both Baldi and Itti (2010) and Maguire et al. (2018) have provided converging theories of surprise which model how people learn from subjectively uncertain outcomes, one based on Bayesian probability and the other on algorithmic information theory.

Whereas classical probability is founded on certainties and fixed causal models, Bayesian probability instead focuses on subjective uncertainties and inductive inference. It extends the classical approach by viewing probability not as an objective phenomenon, but as the state of knowledge of an uncertain observer, a state which is therefore subject to refinement (see Baratgin, 2015). It is an approach that has proved useful for psychological modeling, having been applied to a wide range of cognitive phenomena (e.g., Howson and Urbach, 1991; Pearl, 2001; Chater et al., 2006; Oaksford and Chater, 2007; Darwiche, 2009; Griffiths et al., 2010; Lee, 2011; Tenenbaum et al., 2011; Barber, 2012).

Baldi and Itti (2010) use the Bayesian framework to deliver a model which quantifies the surprisingness or “subjective informativeness” of an observation. Specifically, they suggest that the amount of information that a set of data contains relative to an observer can be measured by the size of the effect the data has on the observer. The amount of learning that has taken place is quantified in terms of the relative entropy (i.e., Kullback-Leibler divergence) between the prior and posterior distributions.

Experimental results have reinforced the value of Baldi and Itti's (2010) surprise notion. For example, they found that it yields a robust performance in predicting human gaze across different spatio-temporal scales, modalities, and levels of abstraction (Itti and Baldi, 2005), while Schauerte and Stiefelhagen (2013) have shown that it can also be applied to detect salient acoustic events.

One issue with Baldi and Itti's (2010) formulation of surprise is that computing it requires identifying a set of relevant hypotheses. Maguire et al. (2018) provide a more generalized theory of surprise based on algorithmic information theory (AIT; see Li and Vitányi, 2008). Whereas Baldi and Itti's (2010) model expresses the informativeness of an observation relative to a pre-defined set of competing hypotheses, Maguire et al.'s model expresses subjective informativeness in terms of the universal likelihood measure of randomness deficiency (see also Maguire et al., 2014, 2016).

Maguire et al.'s (2018) idea is as follows: surprise is the normalised difference between the probabilistic point of view, which treats observations as independent (i.e., it assumes that the uncertainty is objective), and the computational point of view, which gives the shortest possible description of a set of observations (i.e., it allows for the possibility of learning). For example, from the probabilistic point of view the coin tossing outcome string “HHHHHHHHHHHHHHHHHHHH” requires 20 bits to encode, because each bit is independent. However, from the computational points of view the string can be succinctly described in terms of a computer program which says “print 20 Hs.” Accordingly, the outcome of getting 20 heads in a row when tossing an unbiased coin is viewed as surprising. Once we accept that outcomes are not always independent of each other, the pattern of outputs can convey information, leading us, for example, to conclude that a given coin must be biased (see Maguire et al., 2018). The more the probabilistic encoding deviates from the shorter computational encoding, the greater the level of surprise. This framework is consistent with explanation-based accounts of surprise (e.g., Maguire et al., 2011; Foster and Keane, 2015) and AIT-based theories of interestingness (e.g., Dessalles' Simplicity Theory, Dessalles, 2006; Schmidhuber, 2009).

The surprise of a string *x* is a measure of the randomness deficiency of the string relative to the length of its probability-based encoding, i.e., *S*(*x*) = δ(*x*|*p*)/log1/*p*(*x*). It is a normalized value between 0, 1 up to a logarithmic factor. This measure gives us bits of surprise per bit of observation, in other words, the proportion of the probability-based encoding that is superfluous according to the computational point of view. The more an observation can be compressed (i.e., the greater the discrepancy between the probabilistic and computational points of view), the greater the associated level of surprise.

Maguire et al. (2018) show that these two notions of surprise, one Bayesian and the other AIT-based, converge at the limit. Either system can be successfully used to model subjective uncertainty. Nevertheless, Maguire et al. suggest that the AIT approach may prove more amenable to modeling instantaneous “hypothesis-neutral” surprise in open contexts, since it eliminates the requirement of identifying and evaluating a set of competing hypotheses. Instead, the level of surprise is baked into the encoding scheme: the more succinctly the event can be described, the more surprising it is.

### 2.1. Quantifying the likelihood of surprising outcomes

These theories of surprise (Bayesian and AIT-based) allow us to quantify the subjective informativeness of observations, and thus model how people reason about the likelihood of outcomes that could potentially leak subjective information (i.e., surprising outcomes; outcomes that involve learning).

In the case of a surprising lottery sequence such as the Bulgarian example, the two possible explanations, “biased draw” and “coincidence”, are incommensurable. In order to apply probability theory, a single invariant model of reality must be identified, which supports the notion of a repeated experiment (Hohenberg, 2010). But assuming one of these scenarios would be a mistake, because we don't actually know which one is the case.

The problem here is that outcomes can precipitate learning, which alters the underlying probability distributions. Different outcomes cause different learning, resulting in different perspectives, so there is no single stable frame of reference that can be used to compare and contrast potential outcomes (see Zhao and Osherson, 2010; Oaksford and Chater, 2013; Hadjichristidis et al., 2014). Specifically, surprising outcomes alter beliefs about the underlying model, rendering the notion of a repeated experiment inapplicable. Without any objective grounding relative to which the concept of independence can be expressed, there is no agreement on what it is that should be repeated.

One possibility is to use subjective informativeness as a measure of likelihood. As clarified by Shannon (1948), probability and information are the inverse of one another. The likelihood of an event relative to an observer is the inverse of how much information its occurrence would convey to that observer. Shackle (1961) originally proposed that the likelihood of events featuring an element of subjective uncertainty could be quantified in terms of potential surprise, thus establishing an inverse relationship between the concepts of surprise and likelihood (Fisk, 2002; see also Christensen, 1979; Katzner, 1986; Fisk and Pidgeon, 1997; Lagnado and Shanks, 2002; Tentori et al., 2013). Our contribution to the approach of quantifying likelihood in terms of surprisingness is to provide a formalized measure of subjective informativeness. While Baldi and Itti's (2010) measure could equally be used, Maguire et al.'s (2018) quantification is expressed on a scale of 0 to 1, just like classical probability, thus lending itself naturally to such an application.

Quantifying subjective informativeness secures a stable perspective which restores the validity of the frequentist perspective on which appraisals of likelihood depend. The repeated experiment in this case is the act of being surprised. Given the set of all observations, how frequently is it that an observation forces a representational update of this magnitude? In other words, “how frequently does an observation of this type happen to make me this surprised; how frequently does it communicate this amount of information to me personally?” For situations involving asymmetric information, informativeness and likelihood are observer-relative constructs, and differ from person to person.

We contend that most of the reasoning about likelihood that people do in everyday life is based on quantifications of subjective informativeness. Very rarely, except when playing games of chance, do people reason about the relative likelihood of outcomes that involve objective uncertainty.

One interesting difference between judgements involving objective and subjective uncertainty is that they do not follow the same logical rules. In particular, while classical probability, which assumes outcome independence, obeys the conjunction rule, subjective likelihood, which recognizes patterns in outcomes, does not.

Because additional conjunctive events can serve to reduce randomness deficiency, thus increasing subjective likelihood, the addition law of probability, *P*(*A*∪*B*) = *P*(*A*)+*P*(*B*)−*P*(*A*∩*B*), no longer holds. For example, the coin toss pattern *HHHHTT* features less randomness deficiency than *HHHH*, even though the latter is more probable if we assume informational symmetry. The fact that subjective likelihood considers the informational content of an outcome relative to an observer's existing representation (as opposed to “objectively” for all possible observers) adds an additional dimension which undermines its monotonicity.

In the following section we formalize this result by demonstrating that for every situation involving subjective uncertainty there is a conjunction of events which is less surprising (i.e., more subjectively likely) than either of its constituents in isolation.

### 2.2. The conjunction rule does not hold for subjective uncertainty

We prove that given any hypothetical model *p*, there are always two strings of events *x, y*, such that *x* is a substring of *y* but *y* has higher subjective likelihood. The idea of the proof is that any long enough typical string of events can always be decomposed into a substring of events that carries greater subjective information.

**Theorem 1**. Let *E*_1_, *E*_2_, …*E*_*m*_ be *m* independent events, and let *p* be the associated computable probability measure function. Let α>0 be a surprise threshold. There exists a conjunction of events *A* = *A*_1_∧*A*_2_∧…∧*A*_*n*_ with a constituent *B* (i.e., *p*(*A*) < *p*(*B*)) such that *B* is (*p*, α)-surprising (i.e., has a subjective likelihood of < 1) and *A* is (*p*, α)-typical (i.e., has a subjective likelihood of 1).

**Proof**.

Let *E*_1_, *E*_2_, …*E*_*m*_, *p* and α>0 be as above. Without loss of generality *m* = 2^*k*^ and *p* can be seen as a probability on strings of length *k* (each coding one event *E*_*i*_) extended multiplicatively i.e., *p*:2^*k*^ → [0, 1] is extended multiplicatively by *p*(*xy*): = *p*(*x*)*p*(*y*).

Let *n* be a large integer. Let *y*∈2^*kn*^ be a (*p*, α)-typical string. *y* can be viewed as the concatenation of *n* strings of length *k* (i.e., the conjunction of *n* events). By the pigeon hole principle, there must be such a string that occurs at least *n*/2^*k*^ times. Denote this string by *s*, and let *l* be the number of occurrences of *s* in *y*, i.e., *l*≥*n*/2^*k*^. Because *y* is (*p*, α)-typical, we have *p*(*s*)>0. Thus *p*(*s*) = 2^−*c*^ for some *c*>0. Let *x* be *l* concatenations of *s*. Because *p* is extended multiplicatively, we have *p*(*x*)>*p*(*y*).

Let us show that *x* is (*p*, α)-surprising. To describe *x* it suffices to describe *l* plus a few extra bits that say “print *s l* times.” Since *l* can be described in less than 2log*l* bits (by a prefix free program) we have *K*(*x*) < 3log*l* for *n* large enough. We have

$$\begin{array}{lll} -\log p(x)-\alpha & = & -\log p(s^{l})-\alpha =-\log p{\left(s\right)}^{l}-\alpha \\ & = & -l\log2^{-c}-\alpha =cl-\alpha >3\log l>K(x)\\ & \geq & K(x|p^{*}) \end{array}$$

for *n* large enough. Thus *x* is (*p*, α)-surprising, but *y* is not, which ends the proof. The conjunction rule does not hold for subjective likelihood.

In the remainder of the article we investigate whether evidence in support of the so-called conjunction “fallacy” might instead reflect the interpretation by participants of subjective uncertainty in the experimental scenarios. Specifically, we investigate whether rephrasing scenarios to be more objectively uncertain mitigates the conjunction effect, and whether giving participants the time necessary to “learn away” their subjective uncertainty mitigates the conjunction effect.

## 3. Investigating the conjunction effect

Since the 1970s, the dominant view in psychology and behavioral economics has been that humans are prone to making sub-optimal decisions (see Kahneman, 2011). Support for this view depends on the assumption that everyday reasoning should conform to the ideals of probability theory. Where human behavior has been found to deviate from that expectation, participants' decisions have often been interpreted as irrational.

A well-documented example of this supposed irrationality is the conjunction effect, whereby people consistently rate a conjunction as more probable than its constituents in isolation (e.g., Tverksy and Kahneman, 1983; Bar-Hillel and Neter, 1993; Fisk, 2002; Lagnado and Shanks, 2002; Sides et al., 2002; Sloman et al., 2003; Stolarz-Fantino et al., 2003; Tentori et al., 2004; Fisk et al., 2006; Crupi et al., 2008; Nilsson, 2008; Wedell and Moro, 2008; Moro, 2009). Given its salience, the continuing lack of a generally accepted explanation for the conjunction effect is remarkable (Pohl, 2004; Nilsson et al., 2009; Jarvstad and Hahn, 2011).

Nilsson et al. (2009) identify three categories of proposed explanations, namely the representativeness heuristic (Tverksy and Kahneman, 1983; cf. Gavanski and Roskos-Ewoldsen, 1991), misinterpretation of the language used (Hertwig and Gigerenzer, 1999; cf. Tentori et al., 2004; Wedell and Moro, 2008; Tentori and Crupi, 2012), and the effectiveness of heuristics in noisy real-life environments (e.g., Costello, 2009; Juslin et al., 2009).

All of these explanations assume that probability theory provides the normative rules for quantifying likelihood. For instance, even Juslin et al. (2009), who conclude that “the axioms of probability theory may in the end afford little or no benefit at all” (p. 870) still treat the conjunction effect as a fallacy: “This is not to deny the normative status of probability theory. Neither do we claim that it is logical or rational *per se* to commit the conjunction error…it is an intellectual embarrassment” (p. 870).

An alternative approach is to question the appropriateness of probability theory itself in this context (see Oaksford and Chater, 2001). Proponents of a “new paradigm of reasoning” (see Oaksford and Chater, 2007; Over, 2009) argue that logic is inadequate to account for performance in reasoning tasks, as reasoners must use their everyday uncertainty reasoning strategies, whose nature is probabilistic. For example, Cruz et al. (2015) found that participants' decisions were coherent under the assumption that they interpreted natural language conditionals as represented in Bayesian accounts of conditional reasoning, but incoherent under the assumption that they interpreted natural language conditionals in terms of elementary binary logic.

As previously discussed, the use of classical probability assumes that outcomes are independent of each other. While artificial games of chance approach this ideal, virtually all of the reasoning that takes place in everyday life involves subjective uncertainty, where observers learn from their observations and update their representations accordingly. Consequently, we do not believe that classical probability provides the normative rules for quantifying likelihood in the context of everyday human decision-making.

We hypothesize that the scenarios employed by Tverksy and Kahneman (1983) imply subjective uncertainty, thus undermining the applicability of classical probability theory and the conjunction rule. For example, results reported by Tentori et al. (2013) suggest that the added conjunct must be “supportive” of the original proposition, hinting that the conjunction effect may be founded on the reduced surprisingness of the conjunction, an effect they refer to as “inductive confirmation.” When probability and confirmation are disentangled, the latter systematically prevails as a determinant of the conjunction fallacy (see also Politzer and Baratgin, 2016). If a conjunct “confirms” the original proposition, then it renders the original proposition less surprising, suggesting that the effect might be related to surprise. Moreover, Fisk and Pidgeon (1998) found that subjective probability judgements are significantly correlated with judgments of potential surprise for conjunctive outcomes, with a correlation value of −0.90 over a group of 45 statements.

In the following experiment we investigate if rephrasing Tverksy and Kahneman's (1983) classic scenario to reduce the subjective informativeness of the outcomes can mitigate the conjunction effect. Such a finding would suggest that, rather than reasoning irrationally, people are instead correctly applying the mechanics of subjective uncertainty to judge likelihood in situations where they perceive themselves to be at an informational disadvantage.

### 3.1. The linda problem

The most celebrated example of the conjunction effect involves one of the scenarios developed by Tverksy and Kahneman (1983), involving an individual named Linda.

*Linda is 31 years old, single, outspoken, and very bright. She majored in philosophy. As a student, she was deeply concerned with issues of discrimination and social justice, and also participated in anti-nuclear demonstrations*.

1. Which is more probable?
a) Linda is a bank teller
b) Linda is a bank teller and is active in the feminist movement.

Tverksy and Kahneman (1983) reported that, when the two possible outcomes are listed together as above, 85% of people violate the conjunction rule by identifying b) as more probable. Tverksy and Kahneman's explanation of this response is that people get confused by “representativeness.” Participants' responses reflected the extent to which the descriptions matched a stereotype, with a correlation of 0.98 between mean ranks of probability and representativeness. Clearly, representativeness is what people rely on to evaluate likelihood, rather than probability theory (see Tversky and Kahneman, 1974). But is this actually a fallacy?

In the Linda example, some information about Linda is provided, but there is much about her that remains unknown. What kind of person is Linda? Has she settled down since her student days? Participants do not know the answers to these questions, yet the experimenters might, since they are the ones referring to “Linda.” This scenario seems to imply the presence of subjective uncertainty.

If we assume that the description of the Linda scenario is in any way incomplete (e.g., the experimenters know something about her that has not been stated), then the outcomes have the potential to be subjectively informative, meaning that classical probability theory cannot be applied. For example, if we find out that Linda is a bank teller, we might infer that she has become more conservative and has less time for activism, completely altering our understanding of who she is. In contrast, hearing that Linda is still active in the feminist movement suggests that she holds the same beliefs as before. Because these two models of Linda are incommensurable, there is no single objective model relative to which classical probability (i.e., a repeated sample of independent outcomes) can be expressed. The association with representativeness noted by Tverksy and Kahneman (1983) may simply reflect people's use of subjective information for judging likelihood in the face of subjective uncertainty about Linda.

### 3.2. Experiment 1

In order for the Linda scenario to be compatible with classical probability theory, the outcomes must be interpreted as being uninformative, or random, relative to the model of Linda. Instead of being *about* Linda (subjective uncertainty), the uncertainty in the scenario must be *processed by* the Linda (objective uncertainty). This involves interpreting the details provided about “Linda” as being a complete description of a stochastic mechanism that processes randomness.

For example, in a study investigating the 2-4-6 task, Van der Henst et al. (2002) hypothesized that most people fail the task because it is presented in a conversationally misleading way. Specifically, it seems as if the 2-4-6 instance of the rule is a significant one, as opposed to a random one. In order to undermine this interpretation, the experimenters shifted the perspective by making the 2-4-6 appear to emerge from a jackpot machine. Participants thus interpreted 2-4-6 as a random selection from the set of all triples fulfilling the rule, which significantly improved their performance at the task.

In order to achieve a similar shift in perspective, we qualified the original Linda scenario so that the outcomes could be interpreted as independent, random events produced by an unchanging completely specified generative model, like the roll of a dice, or the output of a jackpot machine.

#### 3.2.1. Participants

One hundred and ninety-one undergraduate students from Maynooth University participated voluntarily in this study. These were all second year computer science students who had previously taken introductory mathematics modules in logic, calculus and algebra.

#### 3.2.2. Materials and procedure

The scenario in the *qualified* condition was presented as follows:

*Some programmers run a filter on a large social media database. They input the following randomly selected parameters*.

1) *university_degree* = “*philosophy”*
2) *marital_status* = “*single”*
3) *IQ* > *130*
4) *name* = “*Linda”*
5) *age* = *31*

By chance, a single database record is returned by the filter. Which is more probable?

a) The record states ‘occupation = “bank-teller” and political_outlook = “feminist”’
b) The record states ‘occupation = “bank-teller”’

This qualification of the original scenario is compatible with classical probability theory, because it clearly identifies the description as a completely defined process for selecting a Linda who is a random product of that stochastic model. The outcomes “bank-teller” and “feminist” do not provide any information about the Linda selection filter. All observers share the same information about “Linda,” hence the conjunction rule holds.

The scenario in the *original* condition was presented as follows, so as to be compatible with the information presented in the qualified condition:

*Linda is 31 years old, single, and has an IQ greater than 130. Her university degree was in philosophy*.

Which is more probable?

a) Linda is a bank teller and a feminist
b) Linda is a bank teller

Our hypothesis is that the above description invites the interpretation of subjective uncertainty: Linda is a person, she has more features than those stated in the description, and the outcome provides subjective information about these additional features. Accordingly, neither classical probability nor the conjunction rule hold in this case. Probability is judged based on how subjectively informative the outcomes are. Option a) is less subjectively informative, hence more probable relative to the observer. We therefore hypothesized that the conjunction effect would be observed more frequently in the original condition than in the qualified condition.

Participants were randomly assigned either to the original (*n* = 85) or qualified condition (*n* = 106) and selected one of the two options, which were randomly ordered for each participant. The task was carried out individually on a desktop PC and the results were saved in a database.

### 3.3. Results and discussion

In the original condition, 36% thought it was more probable that Linda was a bank teller, and 64% rated the conjunction as more probable. In the qualified condition, 61% thought it was more probable that the selected Linda would be a bank teller, and 39% rated the conjunction as more probable. A chi-square analysis revealed a significant difference between the two conditions, $\chi_{\left(1\right)}^{2}=11.7$, *p* < 0.001, thus supporting our hypothesis that the conjunction effect would be observed less frequently in the qualified condition.

These results reveal that the exact same description of Linda can be interpreted in a different way when the context in which it has been presented is qualified to suppress the interpretation of leaked subjective information. Without qualification, participants are not sure what or who “Linda” is; they experience subjective uncertainty. If the representation of Linda is subjectively uncertain, then it is not possible to describe the scenario in terms of a fixed stochastic model which enjoys model-outcome independence. Finding out about Linda's occupation changes observers' beliefs about the person, meaning that the presented outcomes are not independent of each other. Probability theory is not applicable in this instance, and the conjunction rule does not hold.

In the qualified condition, the outcomes are random relative to the model, and so the rules of classical probability theory hold. The concept of “Linda” is presented, not as a person, but as the output of a process for selecting a profile on a social media database. In this context, the outcome of whether Linda is a feminist or not tells us nothing about Linda the selection process; the information is clearly objective. An equivalent transform is achieved by Tverksy and Kahneman (1983) when they convert their scenarios into the frequentist perspective by asking participants *how many* individuals selected from a group would match a conjunction of properties. To be clear, when data is presented as frequencies, or as the single output of a stochastic process, then the mechanism is objective, there is no possibility of learning, and the conjunction rule applies. In contrast, Tverksy and Kahneman's original presentation of “Linda” invites the idea of an individual for whom much information is hidden. Because this scenario supports the possibility of learning, the conjunction rule does not hold. We posit that the conjunction fallacy notion which has been developed in the literature has been fueled by a failure to appreciate this difference between objective and subjective uncertainty.

### 3.4. Residual conjunction effect

If subjective uncertainty is eliminated by our qualification of the Linda scenario, the question arises as to why 39% of participants still demonstrated the conjunction effect in this condition. In order to understand this residual anomaly we invited a sample of participants to provide feedback regarding their decision.

Those who rated the conjunction as more likely reported that they thought they were being asked to help decide which of the two outcomes provided the *closest match* for the person to whom the database record belonged. From their perspective, the conjunction appeared less surprising, in other words, more representative of an *ideal* Linda. They viewed the filter as an incomplete description of a real person. In other words, these participants assumed that they were dealing with subjective uncertainty.

Despite our best efforts, a substantial portion of participants still failed to interpret the description of Linda as a complete specification of a selection process. Instead, they continued to think of the description in terms of an incomplete representation of an ideal, mired in subjective uncertainty (i.e., what does Linda look like? what color is her hair? what does she like to eat for breakfast?). Had they interpreted the description as informationally complete, then there would have been no question of some outcomes providing “better” matches than others: every outcome would have been equally uninformative. Instead, they viewed the outcomes as *subjectively informative*, and, correctly given this interpretation, continued to rely on surprisingness rather than probability theory to judge likelihood.

These results illustrate just how counter-intuitive it is to interpret a brief description of a person called Linda as informationally complete. Participants taking part in a psychological experiment expect that the experiment is designed to test some aspect of how they naturally think and behave. Accordingly, it makes sense for them to interpret the description of Linda as if it was a natural everyday situation, presented in the form of text for the sake of convenience. The description of a person called Linda invites participants to think of a particular person who has a personality and other idiosyncratic features, which are known to others, yet subjectively uncertain.

In everyday life it is Linda the person that is relevant, and never Linda the selection mechanism. The form of probability that Tverksy and Kahneman (1983) advocate as representing the reasonable, logical approach to dealing with uncertainty overlooks the ubiquitous presence of subjective uncertainty in everyday situations.

### 3.5. On gambling and when to bet

The idea that a conjunction can be more likely than either of its constituents seems plain wrong. However, Fuchs et al. (2014) point out that all of the rules governing the utility of likelihood judgements are founded on a single requirement, known as Dutch-book coherence. This form of coherence simply requires that an observer's likelihood assignments must never place them in a position where they necessarily suffer a betting loss (Chater et al., 2006).

Clearly, betting on a conjunction will never pay out as often as betting on its constituents in isolation. Subjective likelihood thus appears to violate Dutch-book coherency.

The key to resolving this apparent paradox is that betting only takes place when bettors share the same information. In situations involving asymmetric information, some competitors will hold additional information that gives them an edge, meaning that nobody is going to place a bet. Individuals who are judging probability based on subjective uncertainty do not bet, and do not expose themselves to a monetary loss. They only use the judgment to make decisions for themselves. Indeed, in an empirical study, Maguire et al. (2018) found that, in situations involving subjective uncertainty, using surprisingness to make likelihood judgments leads people to make decisions that optimize their personal success.

When betting is taking place on the toss of a coin there is complete transparency on the nature of the coin, how the coin is going to be tossed and how the call of “heads” or “tails” will be determined. In a fair bet, everyone shares the same objective model of the mechanism that produces the random output, and no outcome is subjectively informative. The outcome of the bet never leads a bettor to update their understanding of how the outcome was produced (or else they would not agree to pay up). Given that there is independence between the model and the outcomes it produces, classical probability theory can be applied.

However, in most everyday situations where likelihood judgements must be made, knowledge of the generative mechanism is not uniform among observers. Typically, people learn from their observations, adjusting their models in light of what they experience. Because some people have access to privileged information, betting is impossible in such situations. Gambling only takes place when participants are confident that everyone in the game experiences the same objective uncertainty.

Tverksy and Kahneman's (1983) Linda scenario does not support betting. People assume that there are aspects of Linda's character that they are not aware of. They are not going to take a bet against the experimenters, because, for all they know, the experimenters might be personal friends of Linda. The Dutch-book coherence supported in cases of asymmetric information is of a “single-user” type, which applies only to observers sharing the same idiosyncratic uncertainty (see Fuchs et al., 2014).

By contrast, the qualified Linda scenario in our experiment supports betting. Here, the Linda scenario is no longer about a particular individual called Linda, but rather constitutes a definitive set of instructions for how a Linda will be selected. Specifically, a Linda will be randomly selected from a social media database to match the description of being 31 years old, single etc. Betting makes sense in this case, as everybody agrees on the model, the outcome is random relative to the model (i.e., nobody has a privileged perspective), and hence no learning takes place. No matter what emerges as the Linda's current occupation, it will not affect the bettor's beliefs about the mechanism by which the Linda was selected.

In sum, probability is the inverse of informativeness. When a situation involves objective uncertainty, then everybody identifies the same probabilities, the conjunction rule holds and betting can take place. However, when information is asymmetric, and subjective uncertainty applies, then different people see different probabilities: outcomes can leak new information, meaning that the conjunction rule no longer holds, and betting does not take place.

### 3.6. Experiment 2

If it was feasible, we would replicate every experiment in Tverksy and Kahneman's (1983) study and, in each case, investigate whether the conjunction effect is mitigated by rephrasing scenarios to support the interpretation of objective uncertainty. However, because most of their experimental scenarios invoke specialized knowledge, they are not amenable to such rephrasing. Ordinary participants are most unlikely to be experts in these specialized areas. Because the scenarios invite participants to interpret themselves as being at an informational disadvantage, they suggest the use of subjective informativeness rather than objective informativeness for evaluating likelihood.

For example, Sides et al. (2002), investigating whether betting instructions would reduce the incidence of the conjunction fallacy, posed once-off questions to participants that invoked specialist knowledge on leukemia vaccines, cigarette taxes, and jury selection procedures. Similarly, Tverksy and Kahneman (1983) posed questions involving geopolitics, medical practice, sports prediction and crime motives. Their scenario involving pulmonary embolism, for instance, was posed to internists taking a physician postgraduate course. Not being experts in the field, these trainees would have interpreted the question as one featuring the potential for learning: how surprised would you be given this outcome; how much would you stand to learn personally from this outcome? Nobody asks a trainee for objective assessment of a tricky question. Instead, trainees get asked questions for teaching purposes. Postgraduates are learning a new skill; they should be expected to treat observations as potentially informative, hence judging probability in terms of subjective informativeness. This isn't a mistake: it simply acknowledges a position of informational disadvantage, with the potential for representational updating to occur following an observation.

Take as another example Tversky and Kahneman's Wimbledon scenario. Participants were asked to predict what would happen in the 1981 final. “Which is more likely,” they were asked “- that Borg will lose the first set, or that he will lose the first set but win the match?” People who bet on sports assume that they know as much about the event as anybody could possibly know, that is, they assume that objective uncertainty applies. This expert knowledge allows professional gamblers to identify value bets. However, sports bettors only reach this stage of objective uncertainty after years of studying a particular game and its athletes. How many of Tverksy and Kahneman's 93 subjects would have felt they knew enough about Bjorn Borg to place a bet on one of his matches? Most likely zero. Hence, those subjects, on being required to make a prediction about the Wimbledon final, undoubtedly viewed themselves as being in a position of informational disadvantage, undermining the applicability of the conjunction rule.

For naive participants, the outcome of Borg losing the first set but winning the match is more likely, because it conveys less subjective information. This is not a fallacy, it is an acknowledgement of not being an expert in sports betting. Because such participants would never dream of placing a bet on a Bjorn Borg match, there is no violation of Dutch-book coherence: the conjunction is *subjectively* more likely; objective probability does not apply. Specifically, if Bjorn Borg lost the match, this would be surprising because Bjorn Borg is supposed to be a great player. If this were to happen, naive observers would have learned something about Bjorn Borg's weaknesses. Given Shannon's observation that information and likelihood are inversely associated concepts, Bjorn Borg losing is viewed as more informative and less likely, as supported by the 72% of participants in Tversky and Kahneman's study who rated it as more probable. In order for the scenario to be interpreted as one involving objective uncertainty, Tverksy and Kahneman's experiment would have to be carried out with seasoned tennis sports bettors. For these professional gamblers, neither outcome is surprising: they've seen it all before and, no matter what happens, they learn nothing new.

The only other experiment in Tverksy and Kahneman's (1983) study that could possibly be construed as involving objective uncertainty is the one involving a dice. Tversky and Kahneman asked participants to consider a dice with four green faces and two red faces. Participants were asked if they would rather bet on

1) RGRRR
2) GRGRRR
or
3) GRRRR

When awarded real payoffs, 65% of participants selected Sequence 2 as most likely, while 62% selected it when the payoffs were merely hypothetical. Sequence 1 can be obtained simply by deleting the first G in sequence 2, therefore, according to the rules of classical probability, the first sequence *must* be more likely. This, of course, assumes that the scenario is interpreted as one involving objective uncertainty. In this regard, there are several mitigating factors.

Firstly, the inclusion of the third sequence, which is highly unrepresentative, suggests that the question is targeting the objectivity of the dice. Its inclusion hints that the sequences of rolls should be evaluated for how much subjective information they convey about the surprising possibility that the dice is biased. Second, it's not obvious that the second sequence is contained in the first. Unless one goes counting and matching letters, it's not obvious that there is a conjunction here. Tverksy and Kahneman (1983) themselves admit that “the relation of inclusion between sequences 1 and 2 was apparently noted by only a few of the subjects.” Finally, few details are provided about how the experiment was carried out. Could participants actually see a dice? Were they able to inspect it? Did they roll it themselves? These are important considerations as regards highlighting the objectivity of the mechanism for deciding the outcome. In sum, it appears there was minimal effort invested in emphasizing to participants that the situation should be interpreted as one involving objective uncertainty.

In the following experiment we replicated Tverksy and Kahneman's (1983) original red-green dice scenario in such a way as to make it completely transparent that objective uncertainty applied. We included only two options, the first relating to a single rolling event, and the other relating to a pair of rolling events. Most importantly, participants were free to repeat the process 30 times, so that any expectations for learning could be fully exhausted, leaving behind a state of clear objective uncertainty.

Hogarth and Soyer (2011) investigated whether a range of classic probability inference errors, including the Linda problem, persist after participants are exposed to a real live version of the problem with which they can experiment. They found that even the statistically naive achieved accurate probabilistic inferences after experiencing sequentially simulated outcomes. In line with Hogarth and Soyer's (2011) findings, our hypothesis is that, as learning is exhausted, the incidence of the conjunction effect should decrease.

#### 3.6.1. Participants

One hundred and fifty-seven undergraduate students from Maynooth University participated voluntarily in this study. These were all second year computer science students who had previously taken introductory mathematics modules in logic, calculus and algebra. None of these students had participated in Experiment 1.

#### 3.6.2. Materials and procedure

Before the process began, participants were introduced to a computer simulated dice with four green faces and two red faces, which they could roll by pressing a button. The experimenter explained that, after making a prediction, participants would roll the dice twice to see if the prediction was correct or not. To incentivise focused decision-making, students were told that the top 20 performers would earn bonus continuous assessment marks. The question was posed as follows:

Which outcome do you wish to gamble on?

a) RED will be rolled second
b) GREEN will be rolled first, and RED will be rolled second

Participants selected an option by clicking a box on the screen. They were also asked to select on a Likert scale “how confident are you that you are playing the game well?.” The dice was then rolled twice and the results displayed on the screen. Participants were invited to repeat this process a total of 30 times, with the cumulative number of correct guesses being tracked.

Participants carried out the task individually on a desktop PC and the results were saved in a database. In the end, all participants with 11 or more correct guesses were awarded the bonus marks, with the top performer achieving 15 correct guesses out of 30.

#### 3.6.3. Results and discussion

In line with Tverksy and Kahneman's (1983) results, there was a notable conjunction effect on the first roll, with 96 out of 157 participants (61%) choosing the conjunction, while the other 61 (39%) gambled on the single red roll. This effect, however, quickly diminished.

In total, the number of participants displaying the conjunction effect dropped from 61% to 29% after 30 trials, with a continuing downward trend. Over the same period, the percentage of participants reporting the highest level of confidence increased from 17% to 30%.

In order to determine whether a trend existed across participants' responses over time, a repeated measures ANOVA was conducted. This revealed a significant difference in responses for the 30 trials, *F*_(29, 4524)_ = 3.666, *p* < 0.001, MSe = 0.172. As expected this data displayed a linear trend, *F*_(1, 156)_ = 29.701, *p* < 0.001, MSe = 0.414, indicating that participants were more likely to exhibit the conjunction fallacy in the initial stages of the experiment than in the latter stages of the experiment.

A similar trend emerged in participants' confidence ratings over time. These differed significantly across the 30 trials *F*_(29, 4524)_ = 3.686, *p* < 0.001, MSe = 0.069. A linear trend was also apparent here, *F*_(1, 156)_ = 13.180, *p* < 0.001, MSe = 0.414, indicating that participants became more confident in their responses over time.

**Figure 1:**
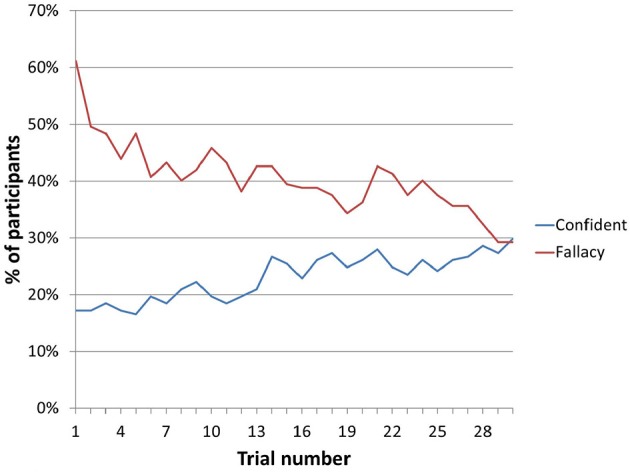
Change over trials in percentage of participants expressing highest level of confidence and demonstrating conjunction fallacy.

Figure 1 shows how the proportion of participants selecting the conjunction continued to fall with successive trials. It also shows how the percentage share of participants selecting the highest level of confidence in their choice continued to grow.

These results show that people quickly adjust to the mechanics of objective uncertainty following a period of learning. Although 61% of participants failed to appreciate the applicability of the conjunction rule based on the initial verbal description at the start of the experiment, they successfully updated their stance once the objectivity of the process was communicated through repeated interaction with the system. In other words, learning about the process by actually carrying it out in practice served to extinguish the conjunction effect.

These results strengthen our claim that Tverksy and Kahneman's (1983) conjunction effect can be explained in terms of learning and subjective information. Figure 1 shows clearly that the proportion of participants demonstrating the fallacy drops over time. This change can only happen if people are changing their opinions in some way, in other words, if they are learning something. Furthermore, the fact that participants' confidence increases over time shows that their uncertainty is of a subjective nature, and not strictly objective: not only are participants learning from the outcomes, but they know that they are learning. Because it assumes independence between outcomes, classical probability excludes the possibility of learning; the conjunction rule therefore does not hold while learning is taking place. As per our hypothesis, the results suggest that as learning becomes exhausted, the incidence of the conjunction effect drops. This implies that the conjunction effect observed here is not a fallacy; instead, it reflects the expectation of learning. The conjunction effect is only a fallacy in cases where participants are certain that they cannot learn anything, and cannot improve their performance at the task any further.

The best way to eliminate subjective uncertainty is to allow people to engage in a judgment task as many times as they want, until they are utterly assured that there is nothing left to be learned. Unfortunately, a hallmark of conjunction fallacy inducing tasks is that they are not repeated: participants are asked a given question a single time, raising the spectre of subjective uncertainty. After 30 trials, nearly a third of our participants were still demonstrating a conjunction effect. Additional research is required to identify whether a true fallacy can ever be observed, or whether the effect would drop to zero with sufficient trials.

Given that it quickly drops off as participants learn about the task, the conjunction fallacy is unlikely to be observed in practice. For example, it is unlikely that professional gamblers could increase their winnings by exploiting the effect. The results of Experiment 2 show that a few seconds of gambling is sufficient for participants to adopt the correct model. Thus, the failure here lies not in people's ability to think rationally. Instead, it lies in the inadequacy of verbal descriptions for communicating objective uncertainty. When people read or hear words, they instead think in terms of subjective uncertainty. Why?

We posit that, rather than exposing a blind spot in human rationality, the conjunction effect simply reflects the unnaturalness of objective uncertainty, especially when communicated via words. Nearly all the decisions that people make in everyday life are based on personal uncertainty, not objective uncertainty. The idea that a diverse set of people might share the same uncertainty is extremely unusual, and only arises when contemplating mechanisms that are capable of manufacturing randomness (perhaps explaining why the mathematical methods of probability were only discovered in the seventeenth century and not earlier). In everyday life, judgments of likelihood, like judgments of informativeness, are nearly always a personal construct (see Fuchs et al., 2014).

The idea that somebody would seek to communicate, through words, the idea of a scenario involving objective uncertainty is especially bizarre. People talk to each other to trade information, hence conversation is dominated by situations involving subjective information. In contrast, people are unlikely to ever discuss situations involving objective randomness, because there is nothing new to learn about such situations. If your friend spent hours talking about unpredictable dice rolls, or objective mechanisms for filtering people on a social media database, you would quickly find yourself a new friend. Verbal descriptions may simply be unsuited to communicating objective uncertainty. Hogarth and Soyer (2011) found that participants preferred to develop models by experimenting with a simulation for themselves rather than accepting a verbal description given by someone else. Further work by Haisley et al. (2010) has shown that experience sampling improves both comprehension and satisfaction with returns in investment decisions that involve risk, suggesting that verbal descriptions often do not succeed in communicating probabilistic models.

It is thus little wonder that people are prone to interpreting Tverksy and Kahneman's (1983) scenarios in terms of subjective uncertainty. Most of these materials make a direct appeal to asymmetric information, focusing on specialized situations in which participants are not expert (e.g., geopolitics, medical practice, sports prediction). The two exceptions in their study are the Linda scenario and the dice scenario. Unfortunately, the Linda scenario leads participants to imagine a person called Linda with whom they are personally unfamiliar, rather than viewing the description as a selection process for women. The only scenario which genuinely supports the interpretation of objective uncertainty is the dice one, and here the objective nature of the uncertainty is disguised by (a) including an unrepresentative sequence as a distractor (b) using long sequences to hide the conjunction and (c) failing to allow participants sufficient interaction with the dice. When these obfuscations are removed, the conjunction effect quickly diminishes.

In sum, based on our results, we believe that previous observations of the conjunction effect reflect, not a deficiency of human logic, but the rational tendency to interpret verbal descriptions in terms of subjective uncertainty (i.e., as if some knowledge is being communicated, and the listener is at an informational disadvantage).

Admittedly, the experiments we have presented in this paper are mere sketches whose results might well be compatible with other competing explanations for the conjunction effect. Further research is required to confirm our theory, specifically focusing on the use of likelihood judgements in ecologically valid settings, where tasks are repeated many times in succession, as they are in real life. The main advantage of our theory is that it has no psychological component: it does not make any assumptions about the idiosyncracies of human behavior. Our theory simply constitutes a mathematical model of how information should be processed in order to optimize decision making in the context of subjective uncertainty; the results of our experiments suggest that people make decisions which reflect the optimal model. Given that our theory is more parsimonious, we suggest that the burden of proof should be placed on those theories that presume that a psychological component is needed to explain the conjunction effect.

## 4. Conclusion

Uncertainty is an intrinsic and omnipresent feature of the real world environment. However, the type of uncertainty we deal with in everyday life is unlike that involved in rolling a dice. We do not assume that our observations are independent of each other. Instead, we know that other people know things that we don't know. We accept the idea that different observers hold different uncertainties, and we watch out for patterns that may be subjectively informative. Accordingly, likelihood judgements in the real world rarely, if ever, obey the conjunction rule.

The notion that the conjunction effect represents a fallacy is firmly entrenched. However, our results suggest that the error is not on the part of the participants, who are sensitive to the subjective informativeness of the outcomes, but on the part of the experimenters, who naively expect classical probability, which assumes objective uncertainty, to apply in real world situations. In everyday life, where subjective uncertainty dominates, it is subjective informativeness rather than classical probability which provides the correct approach for decision-making.

Tverksy and Kahneman (1983) posed the following question: “Why do intelligent and reasonably well-educated people fail to recognize the applicability of the conjunction rule in transparent problems?” In response, we have argued that, for most of Tverksy and Kahneman's scenarios, the conjunction rule does not hold, because participants could potentially learn from the outcomes. And for the one scenario that does involve objective uncertainty, people quickly recognize the applicability of the conjunction rule once they have exhausted the process of learning.

## Ethics statement

These studies, which involved the application of logic and probability, were presented as a component of the module being taught. All subjects participated voluntarily as a learning experience.

## Author contributions

PMa: lead author and experimenter; RM: secondary author and experimenter; PMo: formal theory; MK: general adviser.

### Conflict of interest statement

The authors declare that the research was conducted in the absence of any commercial or financial relationships that could be construed as a potential conflict of interest.
